# Effects of Harvest Maturity, Refrigeration and Blanching Treatments on the Volatile Profiles of Ripe “Tasti-Lee” Tomatoes

**DOI:** 10.3390/foods10081727

**Published:** 2021-07-26

**Authors:** Yu Xi, Qing Li, Jiaqi Yan, Elizabeth Baldwin, Anne Plotto, Erin Rosskopf, Jason C. Hong, Jinhua Zuo, Jinhe Bai, Jian Li

**Affiliations:** 1 Beijing Advanced Innovation Center for Food Nutrition and Human Health, Beijing Technology and Business University, NO. 33 Fucheng Road, Beijing 100048, China; xiyu@btbu.edu.cn (Y.X.); liqing1232021@126.com (Q.L.); zuojinhua@126.com (J.Z.); 2 College of Horticulture, China Agricultural University, NO. 2 Yuanmingyuan West Road, Beijing 100193, China; yanjiaqi@cau.edu.cn; 3 U.S. Horticultural Research Laboratory, 2001 South Rock Road, Ft. Pierce, FL 34945, USA; baldwinliz052@gmail.com (E.B.); anne.plotto@usda.gov (A.P.); erin.rosskopf@usda.gov (E.R.); Jason.hong@usda.gov (J.C.H.); 4 Beijing Key Laboratory of Fruits and Vegetable Storage and Processing, National Engineering Research Center for Vegetables, Beijing Academy of Agriculture and Forestry Sciences, Beijing 100097, China

**Keywords:** *Solanum lycopersicum*, aroma, blanching, chilling, synthetic pathway, volatile, maturity, tomato, flavor, postharvest

## Abstract

The interactive effects of six maturity stages and refrigerated storage (chilling)/blanching (heating) treatments on the volatile profiles of ripe tomatoes were studied. A total of 42 volatiles were identified, of which 19 compounds had odor activity values equal to or greater than 1. Of those, “green” and “leafy” aroma volatiles were most abundant. Chilling and heating treatments both suppressed overall volatile production, with chilling having the greater impact, regardless of harvest maturity. However, fruit harvested at the turning stage had the least volatile suppression by chilling and heating treatments in comparison with fruit harvested earlier or later, mostly in the fatty acid- and phenylalanine-derived volatiles. Volatiles derived from amino acids were promoted by heat treatment for fruit harvested at all maturities, and those derived from carotenoid and phenylalanine pathways and harvested at advanced harvest maturities were stimulated by chilling treatment. Volatile production is generally believed to be improved by delayed harvest, with vine-ripe being optimum. However, opposite results were observed possibly because the later-harvested fruit had longer exposure to open-field weather stress. The best harvest maturity recommendation is the turning stage where fruit developed abundant volatiles and were least impacted by chilling and heating treatments.

## 1. Introduction

Tomato is widely consumed worldwide due to its nutrition, flavor, and processing properties [1]. It has been determined that tomatoes contain large amounts of vitamins, carotene, lycopene, and other antioxidants which are beneficial to human health [2]. Moreover, about 400 volatile compounds have been detected in the ripe tomato that contribute to fruit palatability [3,4,5]. These volatile substances are derived from different pathways such as the amino acid pathway, phenylalanine pathway, fatty acid pathway, and carotenoid pathway [6]. However, many consumers complain that the flavor of modern commercial tomatoes is lacking typical tomato flavor [7]. In fact, the formation of tomato flavor is a complicated process, which is related to many factors, such as variety, pre-harvest environment, and cultural practices; harvest maturity; and postharvest conditions, such as storage temperature, and blanching. In this research we focused on the effect of harvest maturity and postharvest treatments on the volatile profiles of “Tasti-Lee” tomatoes.

Under the current tomato production system, tomatoes are often harvested before full ripeness to avoid postharvest losses and extend storage life [8,9]. Results have not been consistent on how harvest maturity affects the flavor quality of fruit after reaching full red in modern commercial cultivars [5,10,11]. However, it is thought that poor flavor quality often results from harvesting tomatoes prior to breaker stage [8,12]. Xu et al. [13] found that a Florida “heirloom” cultivar had substantially lower quality after reaching full ripe if harvested before the breaker stage, however for commercial cultivars, harvest maturity after mature green did not affect fruit quality in terms of soluble solids content (SSC) and titratable acidity (TA), although differences between harvest maturities for aroma volatiles were detected by an electronic tongue device. “Tasti-Lee” is a hybrid with the crimson gene, high lycopene content, and flavor quality [14,15].

Tomatoes are chilling sensitive at temperatures below 10 °C, and longer exposure time and lower temperature aggravate this sensitivity [16,17]. In Florida, tomatoes are harvested green, gassed with ethylene to initiate and synchronize ripening, then stored at 12–13 °C to slow ripening during packing, repacking, shipping, and marketing [8]. However, on the consumer side, ripe fruit are often stored in the refrigerator (about 5 °C) for several days before cooking and consumption, which has been confirmed to suppress tomato volatiles [18]. However, few studies have been conducted on the interactive effects of the chilling treatment and harvest maturity on tomato flavor.

Blanching (heating) treatment is another method widely used in kitchen and foodservice operations, which can reduce foodborne microorganisms and remove the epidermis structure [19,20,21]. Heating treatment causes volatile flavor loss, which has been reported in many fruits, including in tomatoes [18,22,23], although the volatile production could be partially recovered after a mild high temperature treatment [18,22]. Blanching uses boiling water to treat fruit, and the treated fruit are then immediately cooked or processed. Thus, it is hypothesized there is no time for the fruit to form a visible physiological response after treatment.

The objectives of this research were to analyze the independent and interactive effects of harvest maturity and temperature treatments on volatile profiles of “Tasti-Lee” tomatoes, and to discuss the effects on precursor pathways for aroma volatiles.

## 2. Materials and Methods

### 2.1. Plant Materials

**Figure 1:**
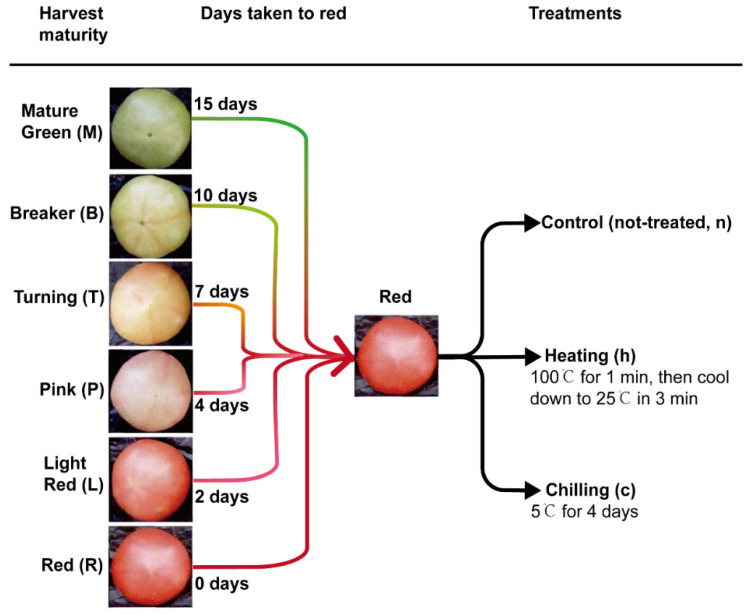
Diagrammatic sketch of overall experimental design. Fruit harvested at six maturities were ripened at 20 °C and then treated by refrigeration (chilling, c), blanching (heating, h), or non-treated (n) as control.

“Tasti-Lee” tomato plants were selected from a tomato research block at the USDA Horticultural Research Laboratory Picos Farm in Fort Pierce, Florida, USA. The harvest maturity of the fruit in the field ranged from immature to full ripe (Figure 1). Sixty fruit of similar size (about 210 g) at each of six mature stages (red, light red, pink, turning, breaker, and mature green) were harvested on 27 December 2015 and stored in a 20 °C dark storage room (humidity: 90%) until they reached the full (red) ripe stage. For each harvest maturity group, when 45 fruits reached red stage (color a* value ≈ 20), the fruits were randomly divided into three temperature treatment sub-groups: non-treated control, refrigeration (chilling; 5 °C air for 4 days), and blanching (heating; 100 °C water for 1 min, then ice water used to cool the fruits to room temperature within 3 min). The changes in core temperature were monitored after blanching. Immediately after the temperature treatment, fruits were further divided into five replicates with three fruits each. The experimental design is depicted in Figure 1.

### 2.2. Sample Preparation

Each replicate sample of three fruits was blended with a homogenizer (Model VM0101, Vita-mix Corp., Cleveland, OH, USA) for one minute. The homogenate, 4.3 g, was pipetted into a 20 mL vial, mixed with 1.7 mL of a saturated CaCl_2_ solution [24], and crimp-capped with a silicone septum (Gerstel Inc., Linthicum, MD, USA). Samples were vortexed, flash frozen in liquid nitrogen, and stored at −80 °C until analysis.

### 2.3. Volatile Analysis

Volatiles were analyzed from the vial headspace using solid-phase micro-extraction, and gas chromatography–mass spectrometry (HS-SPME-GC-MS), as described by Wang et al. [25] with modifications. The volatile substances were extracted using a 2 cm SPME fiber (50/30 μm DVB/Carboxen/PDMS; Supelco, Bellefonte, PA, USA). The homogenate mixture was incubated for 30 min at 40 °C and another 30 min was needed for exposure to the fiber.

The volatile compounds were desorbed in a GC-MS (Model 7890 GC coupled with a 5975 MS detector; Agilent, Santa Clara, CA, USA) by using the HP-5 column (50 m × 0.32 mm × 1.05 μm, J&W Scientific, Agilent, Santa Clara, CA, USA). The GC oven temperature was programmed to increase from 40 to 230 °C at a rate of 4 °C min^−1^ and the holding time was 11.70 min. Helium (37 kPa) was used as the carrier gas at a constant flow of 1.5 mL min^−1^. For the MS system, the temperatures of the inlet, ionizing source, and transfer line were 250, 230, and 280 °C, respectively; electron impact mass units were recorded at 70 eV ionization voltages.

Volatile compounds were tentatively identified by matching their mass spectra to entries in the NIST 14 library (National Institute of Standards and Technology, Gaithersburg, MA, USA) and the retention indexes were compared with the standard volatile compounds. A calibration curve (peak area vs. concentration of reference standards) was prepared from the serial dilutions of the standard and used for sample quantification. Standard aroma compounds (all volatiles identified in this study) were purchased from Sigma-Aldrich (St. Louis, MO, USA) and Fluka Chemical Corporation (Buchs, Switzerland).

### 2.4. Weather Data Collection

Daily maximum and minimum temperatures and precipitation data for 1–14 days before harvest in 2015 were collected from the U.S. Climate Data (Station of Ft. Pierce Florida, 10 km from the farm) (https://www.usclimatedata.com/climate/fort-pierce/florida/united-states/usfl0156, accessed on 10 May 2021).

### 2.5. Statistical Analysis

Data were evaluated by analysis of variance (ANOVA) with the statistical analysis system of SPSS Statistics 17.0 (SPSS Inc., Chicago, IL, USA). Two-way ANOVA was carried out to determine the harvest maturity and treatment interaction (maturity × treatment) on each of the assays. Significant differences across maturity × temperature treatment combined were performed by Duncan’s new multiple range tests, where differences at *p* < 0.05 were considered significant. Principal component analysis (PCA) was performed by Origin 2019b (Microcal Software Inc., Northampton, MA, USA) on the covariance for analyzing the significant differences and relationship of the volatile organic compounds among different treatments.

## 3. Results and Discussion

### 3.1. Characterization of the Volatile Compounds and Effect of Harvest Maturity on Volatile Profiles

A total of 42 volatile compounds were screened by GC-MS in the tomato samples, consisting of 18 aldehydes (methacrolein, butanal, 3-methylbutanal, 2-methylbutanal, 2-methyl-2-butenal, tiglic aldehyde, trans-2-penten-1-al, cis-3-hexenal, hexanal, trans-2-hexenal, heptanal, trans, trans-2, 4-hexadienal, benzaldehyde, octanal, benzeneacetaldehyde, 2-octenal, nonanal, and neral), 4 hydrocarbons (α-pinene, cymene, limonene, and terpinolene), 7 alcohols (2-methylpropanol, 1-penten-3-ol, 3-methylbutanol, 2-methylbutanol, 1-pentanol, 4-methylpentanol, and 3-methylpentanol), 5 ketones (acetone, 2-butanone, 1-penten-3-one, 6-methyl-5-hepten-2-one, and geranyl acetone), 3 oxygen-containing heterocyclic compounds (2-methylfuran, 2-ethyl furan, and 2-pentyl furan), 3 esters (butyl acetate, 2-methylbutyl acetate, and methyl salicylate), 1 sulfur compound (dimethyl disulfide), and 1 sulfur and nitrogen-containing heterocyclic compound (2-isobutylthiazole) (Table 1). The result was similar to the previous report in which 50 aroma volatile compounds were detected by GC-olfactometry in “Tasti-Lee” tomatoes [26]. Aldehydes were the most abundant volatile compounds in the tomatoes regardless of treatment, and the average ratio in the total volatiles was 86.3%, followed by ketones, 9.1%, and alcohols, 3.7%, with the rest each contributing less than 1% (Table 1). For control fruit, aldehydes alone occupied 93.8%, 90.9%, 92.7%, 88.9%, 85.7%, and 93.1% in fruit harvested at red, light red, pink, turning, breaker, and mature green stages, respectively (Table 1 and Table S1). cis-3-Hexenal was the predominant aldehyde among 18 aldehydes in the fruit, constituting 85.4%, 86.6%, 78.4%, 77.4%, 74.8%, and 79.1% of all aldehydes in the tomatoes picked at red, light red, pink, turning, breaker, and mature green stages, respectively (Table 1). The data showed that fruit harvested at the breaker and turning stages had the lowest aldehyde content compared to the earlier (mature green) and later harvest maturities (pink to red); the concentration being higher in mature green fruit (Table 1 and Table S1). However, opposite trends were found for alcohols, ketones, and oxygen-containing heterocyclic compounds. Pink or later harvested fruit had low hydrocarbon concentrations, but earlier harvested fruit had relatively higher concentrations (Table 1 and Table S1). On the other hand, the only sulfur compound, dimethyl disulfide, was not found in the early harvested fruit, but was detected in the pink or later harvested fruit (Table 1 and Table S1).

**Figure 2:**
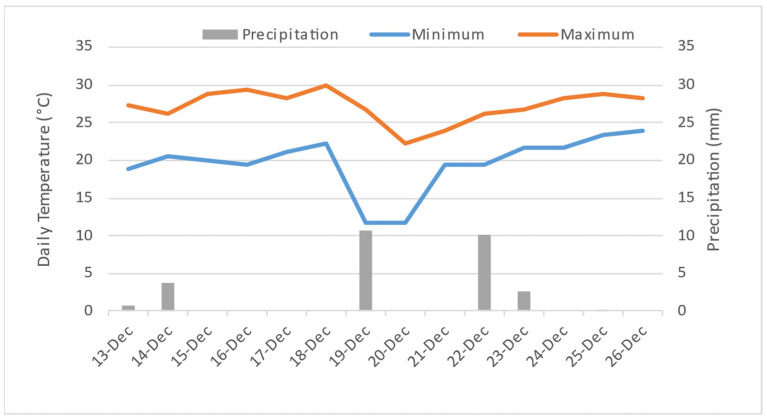
Daily maximum and minimum temperature and precipitation for two weeks before harvest in 2015. Recordings from the U.S. Climate Data (Station of Ft. Pierce Florida, 10 km from the farm) (https://www.usclimatedata.com/climate/fort-pierce/florida/united-states/usfl0156, accessed on 10 May 2021).

Overall flavor quality is generally believed to be better when tomatoes are harvested during later maturity stages, and the best quality fruit are those that are vine-ripened [8,12]. However, different results were observed in this study: the earlier harvested fruit developed more abundant volatiles at the red ripe stage than later-harvested fruit (Table 1 and Table S1). One explanation could be because fruit at turning stage reached the full flavor quality potential, however, the later-harvested fruit had longer exposure to open-field weather stress (Figure 2). Seven and eight days before harvest, fruit experienced two days with low temperatures, 11 °C, which is below chilling injury temperatures in stored fruit [30] (Figure 2). During most of the fruiting season, the minimal air temperature was higher than 18 °C, except on December 19 and 20 when it dropped to 11 °C. This in addition to solar radiation resulted in high temperatures on the fruit surface (Figure 2) [31]. Thus, the pre-harvest low and high temperature stress may negatively affect flavor metabolism, especially for fruit close to being physiologically ripe.

### 3.2. Refrigeration and Blanching Treatment Reduce Volatile Compounds

#### 3.2.1. Refrigeration Treatment

**Figure 3:**
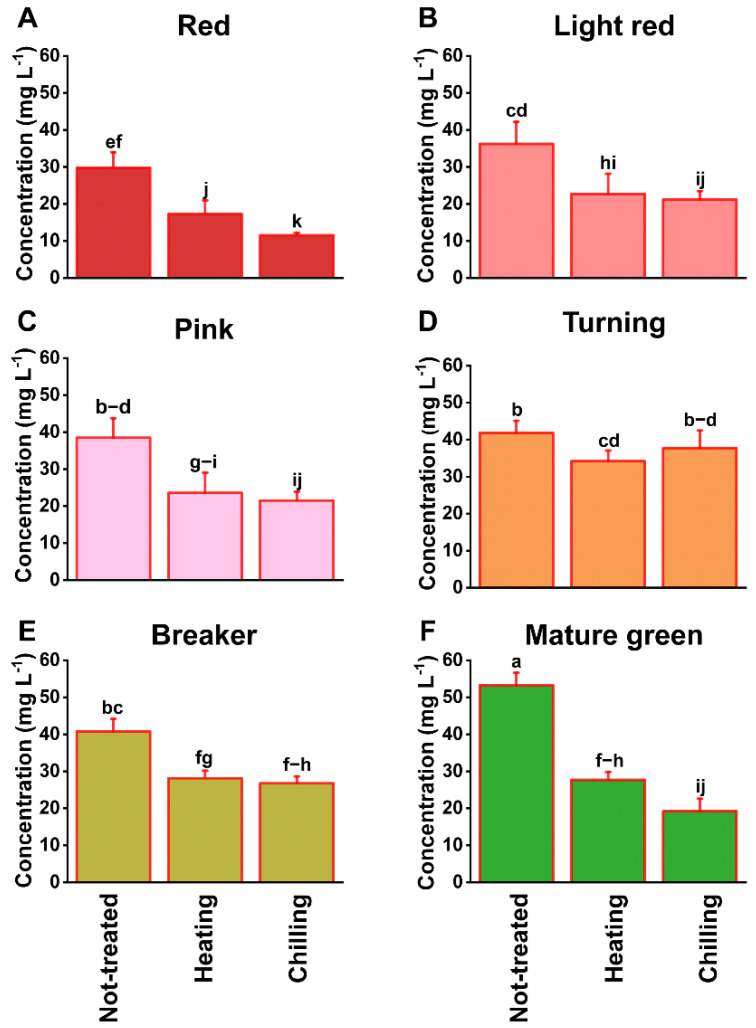
Effect of postharvest temperature treatments on the concentration of flavor compounds (mg L^−1^) in ripe tomatoes harvested at six maturities: (**A**) red, (**B**) light red, (**C**) pink, (**D**) turning, (**E**) breaker, and (**F**) mature green. Each value is the mean of five replicates of three fruits each. Vertical bars represent the standard deviation of the means. The effect of “maturity × treatment” interaction was tested by two-way ANOVA (*p* < 0.05). Different letters on the top of columns represent significant differences between maturity × treatments combined using the Duncan’s multiple range test based on the interaction (*p* < 0.05).

Ripe tomatoes are often stored in a 5–10 °C refrigerator by consumers to extend their shelf life regardless of negative reports that refrigeration suppresses volatile production [18], a form of chilling injury (CI). Although tomatoes are chilling sensitive, full ripe fruit show a lower response to CI [18]. In this experiment, the tomatoes did not express any visual CI symptoms after storage at 5 °C for four days. However, an overall decrease in flavor volatiles was observed in the chilled fruit regardless of harvest maturities (Table 1 and Figure 3). The average volatile loss caused by chilling treatment over all harvest maturities was 42.46% (Table 1, Figure 3, and Table S1). Among those, the highest reduction occurred in the fruit harvested at the mature green stage with 63.82% loss, followed by red (61.20%), pink (43.95%), light red (42.25%), breaker (33.78%), and turning (9.78%) stages (Table 1 and Figure 3), suggesting that the turning and breaker fruits are less sensitive to chilling treatment, and that CI increased along with earlier or later harvests (Figure 3). Among the volatiles, aldehydes, especially cis-3-hexenal, hexanal, and trans-2-hexenal, decreased the most due to chilling treatment (Table 1 and Table S1, and Figure S1). Similar to aldehydes, chilling caused a decrease in esters in all fruit, and alcohols in early harvested fruit and fruit harvested at the red stage (Table 1 and Table S1 and Figure S1). These results are consistent with previous reports that volatile compounds of “FL 47” tomatoes decreased after chilling treatment applied to tomato fruit at the mature green stage, unlike in our study in which chilling treatment was performed when the fruit reached the red-ripe stage [17]. However, it was observed that chilling treatment stimulated some volatile compounds, such as hydrocarbons and oxygen-containing heterocyclic compounds in all materials regardless harvest maturities, and ketones in late harvested fruit (Table 1 and Table S1, and Figure S1). Nevertheless, the concentrations of these compounds were trace, and contributed very little to tomato flavor quality (Figure S1).

#### 3.2.2. Blanching Treatment

Similar to refrigeration, the blanching-treated samples also showed a relative decrease in volatile compounds compared to the control group, although they reduced the volatile concentration through different mechanisms. Refrigeration (chilling) reduced the volatile concentration by slowing and disturbing plant physiological processes, but blanching (heating) reduced volatile concentration mainly by enhanced volatile evaporation/partitioning to the air [18]. The concentration of volatile compounds in blanching-treated tomatoes decreased by 35.4% on average for all harvest maturities (Table 1 and Table S1, and Figure S1). Loss of total volatile compounds was the lowest when the fruits were harvested at the turning stage (Figure 3D), and total volatiles decreased when fruits were harvested at mature/ripe when compared to the turning stage; fruits harvested at mature green lost the most volatiles, 48.00% (Table 1 and Figure 3F). Most of the aldehydes and ketones were reduced by blanching treatment, and some of them, such as cis-3-hexenal, hexanal, trans-2-hexenal, and geranyl acetone, were reduced by approximately 50% (Table 1 and Table S1). This means that even 1 min of blanching, which raised the core temperature of the fruit by about 1 °C on average in this experiment, caused substantial loss of these compounds from surface tissues. However, hydrocarbons, alcohols, and other compounds did not decrease, and some of them even increased (Figure S1B,C,F,G). The high loss ratios of aldehydes and ketones, partially due to low Henry’s law coefficients for these compounds, means that they tend to escape from tissue/solution [32]. This could explain a loss of “freshness” when tomatoes are cooked. A longer cooking time and higher temperature showed significant changes in volatiles, including aldehydes [33].

#### 3.2.3. Comparison of Volatile Profiles between Chilling and Heating Treated Tomatoes

**Figure 4:**
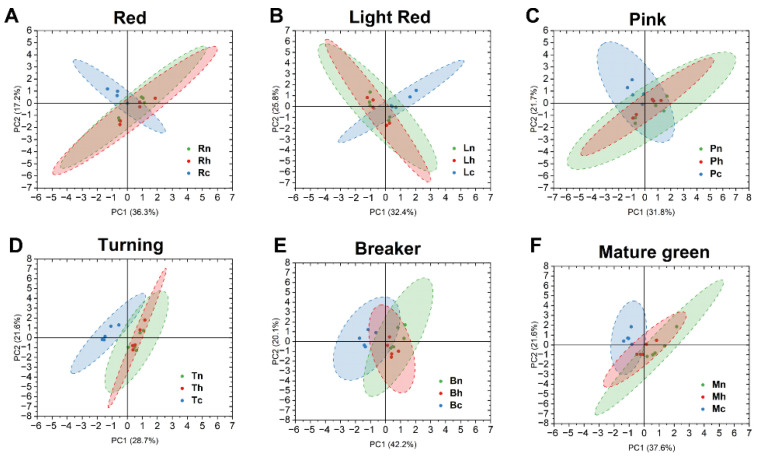
Principal component analysis (PCA) results based on all volatile compounds in “Tasti-Lee” tomatoes with different temperature treatments at six harvest maturities: (**A**) red, (**B**) light red, (**C**) pink, (**D**) turning, (**E**) breaker, and (**F**) mature green. Abbreviations represent combinations of harvest maturity (R—red; L—light red; P—pink; T—turning; B—breaker; and M—mature green) and temperature treatment (h—heating; c—chilling; n—non-treated control).

To compare the volatile profiles of chilling and heating treated tomatoes, a PCA analysis was conducted for all samples harvested at various maturity stages based on all volatile compounds. Generally, heat treatment overlapped with the non-treated control at each harvest maturity, but showed differences to chilling treatment (Figure 4). There were fewer differences in samples harvested at turning and breaker stages, but more for samples harvested at earlier or later maturity (Figure 4). An additional PCA was performed based on 13 selected volatiles (Table 1, marked with #) which are key tomato flavor contributors [11,27,28,29] and represented volatiles from four major pathways: the amino acid pathway, the fatty acid pathway, the phenylalanine pathway, and the carotenoid pathway (Figure S2). Vector loadings showed that high levels of cis-3-hexenal, trans-2-hexenal, hexanal, 1-penten-3-one, and geranyl acetone, abundant acetaldehydes and ketones, were generally associated with the non-treated control. Only one or two compounds, such as 2-methylbutanol, were frequently associated with chilling treatment. All other compounds were often associated with heating and/or the non-treated control (Figure S2).

### 3.3. Response of the Flavor Synthesis Pathways to Refrigeration or Blanching Treatments

#### 3.3.1. Fatty Acid Pathway

**Figure 5:**
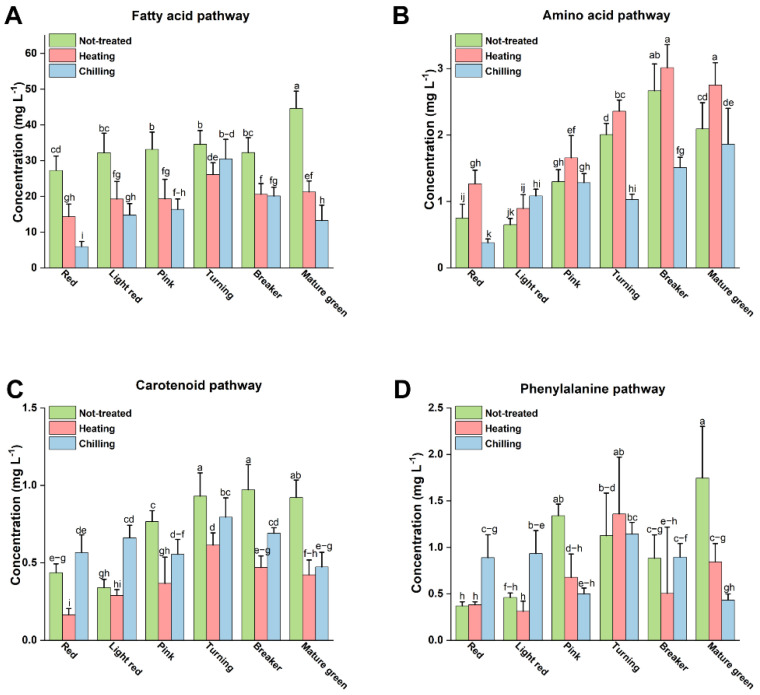
Effects of treatment combinations of harvest maturity and temperature on volatile concentration in tomatoes for aroma volatiles according to their synthesis pathway: (**A**) fatty acid pathway, (**B**) amino acid pathway, (**C**) carotenoid pathway, and (**D**) phenylalanine pathway. Each value is the mean of five replicates. Vertical bars represent standard deviation of the mean. Effect of “maturity × treatment” interaction was tested by two-way ANOVA (*p* < 0.05). Different letters on the top of columns represent significantly differences between treatments using the Duncan’s multiple range test based on the interaction (*p* < 0.05).

The formation of tomato volatile compounds can be categorized into four pathways, including the amino acid pathway, the fatty acid pathway, the phenylalanine pathway, and the carotenoid pathway [5]. Chemicals derived from the fatty acid pathway dominated the volatile compounds regardless of harvest maturity and temperature treatment (Table 1 and Figure S3). The concentrations of fatty acid derivatives substantially decreased after chilling treatment in fruit harvested at all maturities, except at the turning stage in which chilling only caused a small change (Figure 5A). The concentration of three typical C6 aldehydes, cis-3-hexenal, hexanal, and trans-2-hexenal, decreased on average by 50.05%, 38.27%, and 53.65%, respectively, due to chilling treatment (Table 1). At the same time, the concentrations of cis-3-hexenal, hexanal, and trans-2-hexenal in the blanching-treated tomatoes were reduced as well, although by much less in comparison with the chilling treatment (Table 1). Bai et al. [18] reported that in the oxylipin pathway, chilling treatment reduced the above three C6 aldehydes largely due to the downregulation of the gene expression of Tomlox C and HPL, and the activity of hydroperoxide lyase (HPL). HPL is a key enzyme in the fatty acid synthesis pathway and it is easily affected by temperature [34].

#### 3.3.2. Amino Acid Pathway

In tomato fruits, it has been speculated that amino acid derivatives are catalyzed by branched chain aminotransferases (BCATs) to remove amino groups from amino acids [5]. Subsequently, corresponding alcohols are formed after decarboxylation and reduction [35,36]. Isoleucine was a precursor of 2-methylbutanal, while 3-methylbutanol was derived from leucine [5].

Chilling treatment significantly reduced the concentrations of volatile compounds from the amino acid synthesis pathway in most harvest maturities: 11.00%, 43.38%, 48.75%, and 49.58% at mature green, breaker, turning, and red stages, respectively (Table 1, Figure 5B). However, fruit amino acid pathway volatiles, when harvested at the pink stage, were not affected by chilling treatment, and fruit harvested at the light red stage, in contrast, showed amino acid pathway volatiles that increased due to chilling treatment (Table 1, Figure 5B). The result was consistent with the work of Renard et al. [37], which proved that the production of amino acid derivatives decreased after chilling treatment. The reason for chilling-induced increase of amino acid derivatives at the light red stage was not clear.

With high expression of BCATs, there were also high yields of branched volatiles in banana fruits [38], indicating that the reduction of volatile compounds by chilling treatment in this study might be attributed to the decrease of BCATs enzyme activity.

In contrast to chilling treatment, blanching enhanced volatiles from the amino acid pathway (Table 1, Figure 5B). Blanching greatly increased the concentrations of 2-methylbutanal, 3-methylbutanal, 3-methylbutanol, 2-methylbutanol, and 2-isobutylthiazole (Table 1). The concentrations of amino acid pathway volatiles with blanching treatment were increased by 31.55%, 13.03%, 17.50%, 26.91%, 37.86%, and 69.49% at the mature green, breaker, turning, pink, light red, and red stages, respectively (Table 1).

#### 3.3.3. Carotenoid Pathway

In this study, three carotenoid derivatives were detected: neral, 6-methyl-5-hepten-2-one, and geranyl acetone. Among these, the concentration of neral was very low. The amount of two important carotenoid derivatives, 6-methyl-5-hepten-2-one and geranyl acetone, decreased after blanching (Table 1). However, the concentration of 6-methyl-5-hepten-2-one increased after chilling treatment except for fruit harvested at the mature green stage (Table 1), contrary to Klee’s result [36]. On the other hand, the reason for this result may be due to genetic differences between different varieties of tomatoes—both chilling and blanching reduced geranyl acetone concentration (Table 1). Simkin et al. [39] reported that the carotenoid derivatives in tomatoes were associated with genes potentially encoding carotenoid cleavage dioxygenases, LeCCD1A and LeCCD1B. Our results also showed that the carotenoid derivatives were reduced more by chilling or blanching treatment when harvested at early stages (Table 1, Figure 5C).

#### 3.3.4. Phenylalanine Pathway

Two phenylalanine-derived volatiles (methyl salicylate and 2-phenylacetaldehyde) have been detected in this study (Table 1). In this study, no significant changes of methyl salicylate were found, and this might be due to the minimal concentration detected (Table 1). 2-phenylacetaldehyde is reported to be regulated by aromatic amino acid decarboxylases (AADCs), encoded by LeAADC1A, LeAADC1B, and LeAADC2 [40]. Previously, Zou et al. [41] found that the expression of LeAADC1A and LeAADC1B was downregulated during storage at 4 °C. In agreement with Wang et al. [25], the present study also found decreased concentrations of 2-phenylacetaldehyde in chilling injured tomatoes picked at the mature green stage (Table 1, Figure 5D). Intriguingly, here tomato picked at the red and light red stages contained more 2-phenylacetaldehyde when chilling was administered compared to controls, while no significant changes were found in the tomato picked at the breaker or turning stages (Table 1, Figure 5D).

### 3.4. Active Odorants

**Figure 6:**
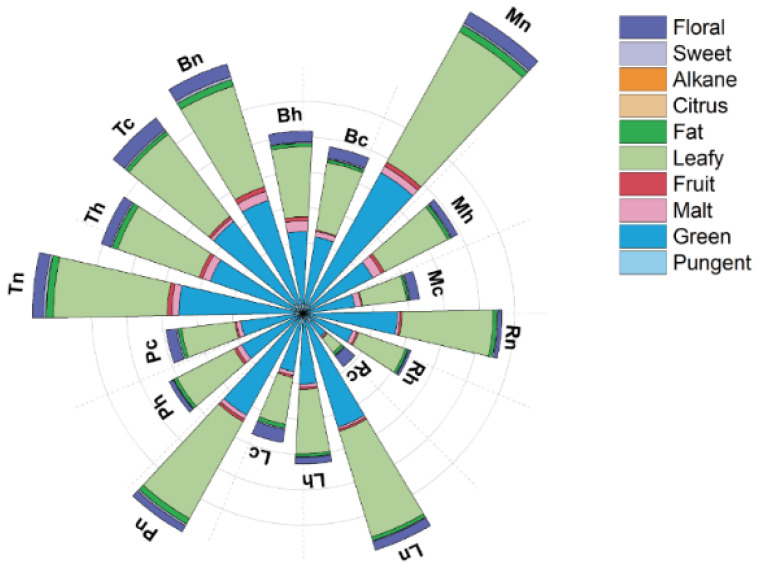
Effects of different treatment combinations of harvest maturity and temperature on aromatic volatiles by reported flavor sensory property (see Table 2). Abbreviations represent combinations of harvest maturity (R—red; L—light red; P—pink; T—turning; B—breaker; and M—mature green) and temperature treatment (h—heating; c—chilling; n—non-treated control).

Based on quantitation, the calculation of odor activity values (OAVs) enables a more reliable evaluation of important odorants in foods [42]. Volatile compounds are considered to contribute to the overall flavor of the tomato when their concentration is greater than detection thresholds [43]. As shown in Table 2, a total of 19 aroma compounds, with their OAVs > 1, were selected. cis-3-Hexenal was the most active compound, which presented “green”, “leafy” notes (Table 2). As shown in the Figure 6, tomatoes mainly emitted the aroma of “green” and “leafy”, confirming that the fatty acid pathway was the important biosynthesis pathway of aroma for “Tasti-Lee” tomatoes. Tomatoes harvested at the mature green stage displayed the strongest “green” and “leafy” notes, as is consistent with many tomato flavor research reports [5,8]. 2-Methylbutanal and 3-methylbutanal were also major contributors to the aroma, which are the derivatives of amino acids and were described as “malt” notes (Table 2) [36]. The aroma of “malt” plays an important part in tomato flavor (Figure 6) [36]. After chilling or blanching treatment, the flavor of tomatoes generally decreased depending on harvest maturity and temperature treatment combinations (Figure 6). Fruit harvested at the turning stage had the second most abundant volatiles after ripening, only less than that in the mature green harvested fruit, and had very limited odor-active volatile loss after chilling and blanching treatments (Table 2, Figure 6).

## 4. Conclusions

The concentration of volatile compounds and OAVs decreased along with advanced maturity in ripened “Tasti-Lee” tomatoes harvested after the turning or later harvest stages. The phenomenon contradicts the traditional understanding that the closer to full ripe at harvest, the better the fruit flavor quality and generally the more abundant the volatile concentrations. Both refrigeration and blanching applied when fruit reached full ripeness (red stage), an approach often used by consumers, substantially reduced the fatty acid derivatives, the dominant volatiles, and the total volatiles, and chilling resulted in greater suppression than blanching. However, the volatiles derived from the amino acid, carotenoid, and phenylalanine pathways showed variable changes related to harvest maturities and chilling/heating treatments. The responses of fruit to refrigeration were based on relatively long-term (4 days) physiological mechanisms and CI, however, responses to blanching were immediate and primarily from the evaporation/partition of the compounds with low Henry’s law coefficient to the atmosphere, such as aldehydes and ketones. Fruit harvested at the turning stage had the highest tolerance to chilling and blanching treatments. This evidence provided in this study offers valuable insight into optimum harvest maturity. Mature green fruit were not able to develop maximum flavor quality based on aroma volatiles. Despite mature green fruit have the most volatile concentration, most of it was “leafy” and “green” cis-3-hexenal. On the other hand, the turning stage, the more advanced maturity, not only developed the best flavor quality (based on volatile profiles), but was also firm enough to resist compression and tough enough to tolerate environmental stress. The assumption that “vine-ripe fruit has the best quality” may mislead growers and consumers, because it is not only fragile to postharvest handling, but also does not have the best flavor quality. Post-ripening refrigeration and blanching both substantially suppress tomato flavor quality at the expense of extending holding time, removing skin and providing sanitation.

## Figures and Tables

**Table 1:** Concentration (mg L^−1^) of volatile compounds determined in ripe tomatoes harvested at six maturity stages and treated with refrigeration (chilling, c), blanching (heating, h), or not-treated (control, n).

			Treatment (Combination of Harvest Maturity and Temperature Treatment)																		ANOVA (Harvest Maturity × Treatment)
	Compounds	RI ^Z^	Rn ^y^	Rh	Rc	Ln	Lh	Lc	Pn	Ph	Pc	Tn	Th	Tc	Bn	Bh	Bc	Mn	Mh	Mc	
**ALDEHYDES**																					
1	Methacrolein	584	0.020 ^d x^	0.027 ^d^	0.100 ^ab^	0.027 ^d^	0.020 ^d^	0.100 ^a^	0.033 ^d^	0.053 ^cd^	0.087 ^bc^	0.091 ^bc^	0.085 ^bc^	0.116 ^ab^	0.091 ^bc^	0.093 ^bc^	0.103 ^ab^	0.117 ^ab^	0.082 ^bc^	0.117 ^ab^	**
2	Butanal	590	0.009 ^g^	0.009 ^g^	0.024 ^ab^	0.008 ^g^	0.009 ^g^	0.019 ^b–d^	0.015 ^c–e^	0.016 ^c–e^	0.02 ^bc^	0.018 ^cd^	0.018 ^cd^	0.015 ^d–f^	0.016 ^c–e^	0.017 ^c–e^	0.019 ^b–d^	0.01 ^fg^	0.012 ^e–g^	0.026 ^a^	**
3	3-Methylbutanal #	638	0.113 ^hi^	0.198 ^fg^	0.091 ^i^	0.138 ^g–i^	0.164 ^gh^	0.176 ^gh^	0.295 ^de^	0.329 ^de^	0.18 ^gh^	0.408 ^bc^	0.558 ^a^	0.203 ^fg^	0.602 ^a^	0.625 ^a^	0.267 ^ef^	0.460 ^b^	0.619 ^a^	0.344 ^cd^	**
4	2-Methylbutanal #	646	0.08 ^gh^	0.146 ^fg^	0.152 ^fg^	0.038 ^h^	0.106 ^gh^	0.264 ^de^	0.391 ^c^	0.447 ^bc^	0.305 ^d^	0.483 ^ab^	0.539 ^a^	0.212 ^ef^	0.534 ^a^	0.495 ^ab^	0.304 ^d^	0.395 ^c^	0.421 ^bc^	0.405 ^c^	**
5	2-Methyl-2-butenal	719	0.028 ^de^	0.041 ^d^	- ^w^	0.010 ^e^	0.036 ^d^	-	0.076 ^c^	0.089 ^bc^	-	0.104 ^b^	0.089 ^bc^	-	0.125 ^a^	0.130 ^a^	-	0.084 ^bc^	0.099 ^b^	-	**
6	Tiglic aldehyde	1101	0.111 ^e^	0.164 ^e^	-	0.041 ^f^	0.143 ^e^	-	0.303 ^d^	0.357 ^cd^	-	0.414 ^b^	0.357 ^cd^	-	0.499 ^a^	0.520 ^a^	-	0.336 ^d^	0.394 ^bc^	-	**
7	*trans*-Penten-1-al	1131	0.136 ^c^	0.107 ^cd^	0.023 ^f^	0.210 ^b^	0.140 ^c^	0.092 ^d^	0.137 ^c^	0.081 ^de^	0.084 ^de^	0.221 ^b^	0.211 ^b^	0.230 ^b^	0.242 ^b^	0.098 ^cd^	0.079 ^de^	0.312 ^a^	0.100 ^cd^	0.042 ^ef^	**
8	*cis*-3-Hexenal #	771	23.833 ^bc^	12.681 ^f^	4.485 ^g^	28.839 ^b^	16.613 ^d–f^	11.931 ^f^	27.966 ^b^	16.269 ^d–f^	13.428 ^ef^	28.782 ^b^	21.748 ^cd^	26.528 ^bc^	26.017 ^bc^	17.851 ^de^	17.993 ^de^	37.889 ^a^	18.179 ^de^	11.427 ^f^	**
9	Hexanal #	774	1.113 ^d^	0.784 ^ef^	0.892 ^d–f^	1.045 ^de^	0.813 ^ef^	1.075 ^de^	1.711 ^bc^	1.017 ^de^	1.013 ^de^	1.973 ^ab^	1.553 ^c^	1.079 ^de^	1.896 ^ab^	0.965 ^d–f^	0.774 ^ef^	2.045 ^a^	0.964 ^d–f^	0.670 ^f^	**
10	*trans*-2-Hexenal #	828	2.056 ^de^	1.339 ^gh^	0.503 ^i^	2.446 ^cd^	1.588 ^e–h^	1.540 ^e–h^	3.340 ^b^	1.853 ^e–g^	1.766 ^e–g^	3.535 ^b^	2.691 ^c^	2.625 ^c^	3.852 ^b^	1.999 ^d–f^	1.450 ^f–h^	4.413 ^a^	1.936 ^d–f^	1.142 ^h^	**
11	Heptanal	875	0.008 ^c^	0.009 ^c^	0.011 ^c^	0.007 ^cd^	0.003 ^d^	0.015 ^bc^	0.014 ^bc^	0.010 ^c^	0.010 ^c^	0.017 ^b^	0.014 ^bc^	0.014 ^bc^	0.016 ^b^	0.008 ^c^	0.010 ^c^	0.025 ^a^	0.010 ^c^	0.009 ^c^	**
12	*trans, trans*-2, 4-Hexadienal	887	-	-	-	-	-	-	-	-	-	-	0.613 ^a^	-	-	-	-	-	-	-	**
13	Benzaldehyde	945	0.010 ^bc^	0.006 ^de^	0.002 ^ef^	0.010 ^bc^	0.003 ^d–f^	0.007 ^cd^	0.011 ^b^	0.004 ^d–f^	0.002 ^ef^	0.015 ^a^	0.01 ^bc^	0.006 ^de^	0.011 ^b^	0.001 ^f^	0.002 ^ef^	0.018 ^a^	0.003 ^d–f^	0.002 ^ef^	**
14	Octanal	969	0.001 ^b^	0.002 ^b^	0.001 ^b^	0.001 ^b^	-	0.002 ^b^	-	0.001 ^b^	0.004 ^a^	-	0.001 ^b^	0.001 ^b^	-	-	0.001 ^b^	0.001 ^b^	-	-	**
15	2-phenylacetaldehyde	1049	0.366 ^i^	0.375 ^i^	0.887 ^c–h^	0.444 ^g–i^	0.296 ^i^	0.93 ^b–f^	1.334 ^a–c^	0.674 ^e–i^	0.499 ^f–i^	1.125 ^b–e^	1.31 ^ab^	1.148 ^b–d^	0.859 ^c–h^	0.504 ^f–i^	0.865 ^c–g^	1.742 ^a^	0.844 ^d–h^	0.437 ^hi^	**
16	2-Octenal	1074	0.032 ^ab^	0.038 ^a^	-	0.037 ^a^	0.003 ^c^	-	0.034 ^a^	-	-	-	0.014 ^bc^	-	-	-	-	0.034 ^a^	-	-	**
17	Nonanal	1059	-	-	-	0.001 ^a^	-	-	-	-	-	-	-	-	-	-	-	-	-	-	**
18	Neral #	1180	0.006	-	0.004	-	0.005	-	0.009	0.005	-	-	-	-	-	-	-	0.010	-	-	NS
**HYDROCARBONS**																					
1	α-Pinene	910	-	0.001	-	-	-	-	-	-	0.001	-	-	0.001	-	-	0.001	-	-	0.001	NS
2	Cymene	994	-	-	-	-	-	-	-	-	-	-	-	0.001	-	-	0.001	-	-	0.001	NS
3	Limonene	998	0.024 ^e–g^	0.005 ^g^	0.068 ^d^	0.009 ^fg^	0.012 ^fg^	0.077 ^d^	0.007 ^fg^	0.011 ^fg^	0.06 ^de^	0.050 ^d–f^	0.116 ^c^	0.342 ^a^	0.062 ^de^	0.044 ^d–g^	0.330 ^a^	0.084 ^cd^	0.123 ^c^	0.281 ^b^	**
4	Terpinolene	1048	-	-	-	-	-	-	-	-	-	-	-	0.001 ^a^	-	-	0.001 ^a^	-	-	-	**
**ALCOHOLS**																					
1	2-Methylpropanol	612	0.006 ^f^	0.018 ^de^	-	-	0.014 ^ef^	0.019 ^c–e^	0.017 ^de^	0.022 ^c–e^	0.067 ^a^	0.033 ^b–d^	0.020 ^c–e^	0.009 ^ef^	0.035 ^bc^	0.048 ^b^	0.014 ^ef^	0.005 ^ef^	0.062 ^a^	0.076 ^a^	**
2	1-Penten-3-ol	1157	0.055 ^d^	0.031 ^e^	0.028 ^e^	0.085 ^b^	0.067 ^b–d^	0.067 ^b–d^	0.034 ^e^	0.030 ^e^	0.054 ^d^	0.085 ^b^	0.073 ^b–d^	0.083 ^bc^	0.086 ^b^	0.057 ^d^	-	0.109 ^a^	0.065 ^cd^	-	**
3	3-Methylbutanol #	707	0.365 ^de^	0.656 ^cd^	0.068 ^g^	0.329 ^d–f^	0.379 ^de^	0.222 ^ef^	0.315 ^d–f^	0.443 ^d^	0.183 ^fg^	0.604 ^d^	0.767 ^c^	0.296 ^d–f^	0.927 ^b^	1.189 ^a^	0.368 ^de^	0.770 ^c^	1.106 ^a^	0.350 ^de^	**
4	2-Methylbutanol #	711	0.190 ^g–i^	0.270 ^gh^	0.067 ^i^	0.144 ^hi^	0.244 ^gh^	0.417 ^ef^	0.292 ^fg^	0.424 ^ef^	0.613 ^bc^	0.508 ^c–e^	0.489 ^c–e^	0.313 ^fg^	0.599 ^b–d^	0.703 ^ab^	0.567 ^b–d^	0.464 ^de^	0.606 ^b–d^	0.761 ^a^	**
5	1-Pentanol	775	0.066 ^b^	0.091 ^a^	0.021 ^ef^	0.049 ^bc^	0.051 ^bc^	0.026 ^d–f^	0.009 ^fg^	0.044 ^cd^	0.025 ^def^	0.052 ^bc^	0.042 ^c–e^	0.026 ^d–f^	0.057 ^bc^	0.041 ^c–e^	0.016 ^f^	0.052 ^bc^	0.022 ^ef^	-	**
6	4-Methylpentanol	809	-	0.003 ^b^	0.005 ^b^	0.002 ^b^	0.003 ^b^	-	-	-	-	-	-	-	0.009 ^b^	0.039 ^a^	-	0.009 ^b^	0.031 ^a^	-	**
7	3-Methylpentanol	817	0.015 ^d–f^	0.013 ^f^	0.011 ^f^	0.015 ^d–f^	0.013 ^ef^	0.023 ^bc^	0.014 ^ef^	0.015 ^d–f^	0.023 ^bc^	0.018 ^c–e^	0.020 ^b–d^	0.013 ^ef^	0.030 ^a^	0.023 ^bc^	0.013 ^ef^	0.025 ^ab^	0.019 ^c–e^	0.019 ^c–e^	**
**KETONES**																					
1	Acetone	533	0.423 ^f^	0.453 ^f^	3.090 ^ab^	2.004 ^d^	1.363 ^e^	2.939 ^a–c^	1.096 ^e^	0.727 ^ef^	2.335 ^cd^	1.998 ^d^	1.988 ^d^	3.106 ^a^	2.569 ^a–d^	2.389 ^cd^	2.45 ^b–d^	2.447 ^a–d^	1.285 ^e^	2.171 ^d^	**
2	2-Butanone	591	0.002 ^b^	0.013 ^a^	-	-	-	0.003 ^b^	-	-	-	-	-	-	-	-	-	-	-	-	**
3	1-Penten-3-one #	665	0.197 ^b–d^	0.124 ^ef^	0.071 ^f^	0.253 ^ab^	0.232 ^a–c^	0.172 ^c–e^	0.139 ^de^	0.147 ^de^	0.129 ^ef^	0.244 ^ab^	0.203 ^a–d^	0.230 ^a–c^	0.265 ^a^	0.175 ^c–e^	0.008 ^g^	0.255 ^ab^	0.146 ^de^	0.005 ^g^	**
4	6-Methyl-5-hepten-2-one #	950	0.084 ^i^	0.078 ^i^	0.279 ^a–e^	0.107 ^hi^	0.082 ^i^	0.329 ^a^	0.204 ^e–g^	0.161 ^gh^	0.248 ^c–f^	0.254 ^b–f^	0.205 ^e–g^	0.304 ^a–c^	0.216 ^d–g^	0.197 ^fg^	0.287 ^a–d^	0.317 ^ab^	0.190 ^fg^	0.247 ^c–f^	**
5	Geranyl acetone #	1367	0.345 ^ef^	0.084 ^h^	0.295 ^e–g^	0.233 ^fg^	0.203 ^g^	0.332 ^ef^	0.553 ^c^	0.201 ^g^	0.307 ^e–g^	0.676 ^ab^	0.401 ^de^	0.491 ^cd^	0.753 ^a^	0.271 ^fg^	0.408 ^de^	0.592 ^bc^	0.232 ^fg^	0.227 ^fg^	**
**OXYGEN-CONTAINING HETEROCYCLIC COMPOUNDS**																					
1	2-Methylfuran	602	0.006 ^f^	0.011 ^ef^	0.062 ^a^	0.005 ^f^	0.009 ^ef^	0.064 ^a^	0.010 ^ef^	0.014 ^d–f^	0.030 ^b^	0.013 ^d–f^	0.016 ^de^	0.024 ^bc^	0.008 ^ef^	0.011 ^ef^	0.024 ^bc^	0.014 ^d–f^	0.007 ^f^	0.020 ^cd^	**
2	2-Ethyl furan	676	0.014 ^b–d^	0.010 ^d–f^	0.010 ^c–f^	0.016 ^b^	0.011 ^b–f^	0.015 ^b–d^	0.014 ^b–e^	0.008 ^f^	0.010 ^c–f^	0.023 ^a^	0.021 ^a^	0.015 ^bc^	0.023 ^a^	0.014 ^b–e^	0.009 ^ef^	0.027 ^a^	0.014 ^b–e^	0.006 ^f^	**
3	2-Pentyl-furan	996	0.054 ^d–f^	0.056 ^d–f^	0.267 ^b^	0.057 ^d–f^	0.034 ^ef^	0.256 ^b^	0.088 ^d^	0.080 ^d^	0.074 ^de^	0.064 ^de^	0.045 ^d–f^	0.298 ^b^	0.145 ^c^	0.016 ^f^	0.395 ^a^	0.133 ^c^	0.080 ^d^	0.401 ^a^	**
**ESTERS**																					
1	Butyl acetate	774	0.001 ^c^	0.003 ^bc^	0.004 ^ab^	0.002 ^c^	0.002 ^c^	0.003 ^c^	0.005 ^a^	0.004 ^ab^	0.001 ^c^	0.003 ^bc^	0.005 ^ab^	0.001 ^a^	0.003 ^bc^	0.003 ^bc^	-	0.003 ^bc^	0.004 ^ab^	0.001 ^c^	**
2	2-Methylbutyl acetate	847	-	-	-	-	-	-	-	0.001 ^a^	-	-	0.001 ^a^	-	-	0.001 ^a^	-	0.001 ^a^	0.001 ^a^	-	**
3	Methyl salicylate #	1156	0.003	0.009	-	0.011	0.013	-	0.004	0.001 ^c^	0.002	0.005	0.003	-	-	-	-	-	-	-	NS
**SULFUR- AND NITROGEN-CONTAINING HETEROCYCLIC COMPOUNDS**																					
1	2-Isobutylthiazole #	1002	0.002 ^de^	0.003 ^b–e^	-	0.001 ^e^	0.001 ^e^	0.002 ^c–e^	0.004 ^a–c^	0.004 ^a–c^	0.003 ^b–e^	0.004 ^a–c^	0.006 ^a^	0.005 ^a^	0.004 ^a–c^	0.002 ^de^	0.004 ^a–c^	0.003 ^b–e^	0.001 ^e^	0.003 ^b–e^	**
**SULFUR COMPOUNDS**																					
1	Disulfide, dimethyl	752	0.001 ^b^	0.001 ^b^	0.001 ^b^	0.001 ^b^	0.001 ^b^	0.001 ^b^	0.001 ^b^	-	-	-	-	0.004 ^a^	-	-	-	-	-	-	**
**TOTAL VOLATILES**			29.78 ^ef^	17.86 ^j^	11.52 ^k^	36.63 ^cd^	22.68 ^hi^	21.12 ^ij^	38.48 ^b–d^	23.54 ^ghi^	21.56 ^ij^	41.82 ^b^	34.23 ^de^	37.74 ^b–d^	40.55 ^bc^	28.53 ^fg^	26.76 ^f–h^	51.44 ^a^	29.42 ^f–g^	19.19 ^ij^	**

^z^: RI = retention index on a HP-5 column (Agilent). ^y^: Abbreviations of combination of harvest maturity (R—red; L—light red; P—pink; T—turning; B—breaker; and M—mature green) and temperature treatment (h—heating; c—chilling; and n—non-treated control). ^x^: Means followed by different superscripts in the same row indicate significant differences using the Duncan’s multiple range test (*p* < 0.05). ^w^: Not detectable. ** *p* < 0.001. NS: not significant. ^#^ 13 important volatiles which are deemed key tomato volatiles [11,27,28,29] and were selected for PCA analysis.

**Table 2:** Odor activity values (OAVs) of active volatile compounds determined in the different treated tomatoes harvested at six maturity stages and treated by refrigeration (chilling, c), blanching (heating, h), or non-treated (control, n).

Compounds		Odor Threshold in Water (mg L^−1^) ^Z^	Odor Description ^Y^	Treatment (Combination of Harvest Maturity and Temperature Treatment)																	
				Rn ^x^	Rh	Rc	Ln	Lh	Lc	Pn	Ph	Pc	Tn	Th	Tc	Bn	Bh	Bc	Mn	Mh	Mc
**ALDEHYDES**																					
1	Butanal	0.0090	Pungent, green	1.0	1.0	2.7	0.9	1.0	2.1	1.7	1.8	2.2	2.0	2.0	1.7	1.8	1.9	2.1	1.1	1.3	2.9
2	3-Methylbutanal	0.0002	Malt	753.3	1320.0	606.7	920.0	1093.3	1173.3	1966.7	2193.3	1200.0	2720.0	3720.0	1353.3	4013.3	4166.7	1780.0	3066.7	4126.7	2293.3
3	2-Methylbutanal	0.0030	Cocoa, almond, malt	27.0	48.7	50.7	12.7	35.3	88.0	130.3	149.0	101.7	161.0	179.7	70.7	178.0	165.0	101.3	131.7	140.3	135.0
4	trans-Pentenal-1-al	0.0892	Strawberry, fruit, tomato	1.5	1.2	0.3	2.4	1.6	1.0	1.5	0.9	0.9	2.5	2.4	2.6	2.7	1.1	0.9	3.5	1.1	0.5
5	cis-3-Hexenal	0.0003	Leafy, green	95,332.0	50,724.0	17,940.0	115,356.0	66,452.0	47,724.0	111,864.0	65,076.0	53,712.0	115,128.0	86,992.0	106,112.0	104,068.0	71,404.0	71,972.0	151,556.0	72,716.0	45,708.0
6	Hexanal	0.0045	Grass, tallow, fat	247.3	174.2	198.2	232.2	180.7	238.9	380.2	226.0	225.1	438.4	345.1	239.8	421.3	214.4	172.0	454.4	214.2	148.9
7	trans-2-Hexenal	0.0170	Green, leafy	120.9	78.8	29.6	143.9	93.4	90.6	196.5	109.0	103.9	207.9	158.3	154.4	226.6	117.6	85.3	259.6	113.9	67.2
8	Heptanal	0.0030	Fat, citrus, rancid	2.7	3.0	3.7	2.3	1.0	5.0	4.7	3.3	3.3	5.7	4.7	4.7	5.3	2.7	3.3	8.3	3.3	3.0
9	Octanal	0.0007	Fat, soap, lemon, green	1.4	2.9	1.4	1.4	0.0	2.9	0.0	1.4	5.7	0.0	1.4	1.4	0.0	#VALUE!	1.4	1.4	0.0	0.0
10	2-phenylacetaldehyde	0.0063	Hawthome, sweet, honey	58.1	59.5	140.8	70.5	47.0	147.6	211.7	107.0	79.2	178.6	207.9	182.2	136.3	80.0	137.3	276.5	134.0	69.4
11	2-Octenal	0.0030	Green, nut, fat	10.7	12.7	0.0	12.3	1.0	0.0	11.3	0.0	0.0	0.0	4.7	0.0	0.0	0.0	0.0	11.3	0.0	0.0
**HYDROCARBONS**																					
1	Limonene	0.0100	Lemon, orange	2.4	0.5	6.8	0.9	1.2	7.7	3.4	0.0	0.0	5.0	11.6	34.2	6.2	4.4	33.0	8.4	12.3	28.1
**ALCOHOLS**																					
1	3-Methylbutanol	0.2500	Whiskey, malt, burnt	1.5	2.6	0.3	1.3	1.5	0.9	0.1	0.0	0.0	2.4	3.1	1.2	3.7	4.8	1.5	3.1	4.4	1.4
2	2-Methylbutanol	0.2500	Malt, wine, onion	0.8	1.1	0.3	0.6	1.0	1.7	0.1	0.0	0.0	2.0	2.0	1.3	2.4	2.8	2.3	1.9	2.4	3.0
**KETONES**																					
1	1-Penten-3-one	0.0015	Fruity, floral, green	131.3	82.7	47.3	168.7	154.7	114.7	22.7	0.0	0.0	162.7	135.3	153.3	176.7	116.7	5.3	170.0	97.3	3.3
2	6-Methyl-5-hepten-2-one	0.0500	Fruity, floral	1.7	1.6	5.6	2.1	1.6	6.6	0.7	0.0	0.0	5.1	4.1	6.1	4.3	3.9	5.7	6.3	3.8	4.9
3	Geranyl acetone	0.0600	Sweet, floral, estery	5.8	1.4	4.9	3.9	3.4	5.5	0.6	0.0	0.0	11.3	6.7	8.2	12.6	4.5	6.8	9.9	3.9	3.8
**OXYGEN-CONTAINING HETEROCYCLIC COMPOUNDS**																					
1	2-Pentyl- furan	0.0058		9.3	9.7	46.0	9.8	5.9	44.1	5.9	0.0	0.0	11.0	7.8	51.4	25.0	2.8	68.1	22.9	13.8	69.1
**SULFUR- AND NITROGEN-CONTAINING HETEROCYCLIC COMPOUNDS**																					
1	2-Isobutylthiazole	0.0035	Tomato leafy, green	0.6	0.9	0.0	0.3	0.3	0.6	9.7	0.0	0.0	1.1	1.7	1.4	1.1	0.6	1.1	0.9	0.3	0.9

^z^: Odor description of 6-methyl-5-hepten-2-one adapted from Klee [36], while others from Acree and Arn [44]. ^y^: Odor threshold values (detection) in water adapted from Van Gemert [45]. When there was more than one value, those from the USDA, ARS laboratory in Albany (CA) were chosen for consistency of the methodology. ^x^: Abbreviations of combination of harvest maturity (R—red; L—light red; P—pink; T—turning; B—breaker; and M—mature green) and temperature treatment (h—heating; c—chilling; n—non-treated control).

## Data Availability

The data presented in this study are available in article and Supplementary Materials.

## Supplementary Materials

The following are available online at https://www.mdpi.com/article/10.3390/foods10081727/s1, Figure S1: Effects of treatment combinations of harvest maturity and temperature on volatile concentration in tomatoes by chemical class: (A) aldehydes, (B) hydrocarbons, (C) alcohols, (D) ketones, (E) oxygen-containing heterocyclic compounds, (F) esters, (G) sulfur- and nitrogen-containing heterocyclic compounds, and (H) sulfur compounds. Each value is the mean of five replicates. Vertical bars represent standard deviation of the mean. The effect of the “maturity × treatment” interaction was tested by two-way ANOVA (*p* < 0.05). Different letters on the top of columns represent significantly differences between treatments using the Duncan’s multiple range test (*p* < 0.05). Figure S2: Principal component analysis (PCA) results based on the 13 key volatile substances in “Tasti-Lee” tomatoes with different temperature treatments by six harvest maturities: (A) red, (B) light red, (C) pink, (D) turning, (E) breaker, and (F) mature green. Abbreviations represent combinations of harvest maturity (R—red; L—light red; P—pink; T—turning; B—breaker; and M—mature green) and temperature treatment (h—heating; c—chilling; n—non-treated control). Figure S3: Total volatile concentrations of all treatment combinations of harvest maturity and temperature treatment of tomatoes by synthesis pathways. Abbreviations represent combinations of harvest maturity (R—red; L—light red; P—pink; T—turning; B—breaker; and M—mature green) and temperature treatment (h—heating; c—chilling; n—non-treated control). Table S1: Relative concentration (%) of each chemical class determined in tomatoes harvested at six maturity stages and treated by refrigeration (chilling, c), blanching (heating, h), or non-treated control.
