# Supplementary material for: Effects of Harvest Maturity, Refrigeration and Blanching Treatments on the Volatile Profiles of Ripe “Tasti-Lee” Tomatoes
Source: Foods. 2021 Jul 26;10(8):1727. doi: 10.3390/foods10081727 (PMC8391134; doi:10.3390/foods10081727)
Supplement: Supplementary file 1 [file foods-10-01727-s001.zip › Fighure S1.pdf]

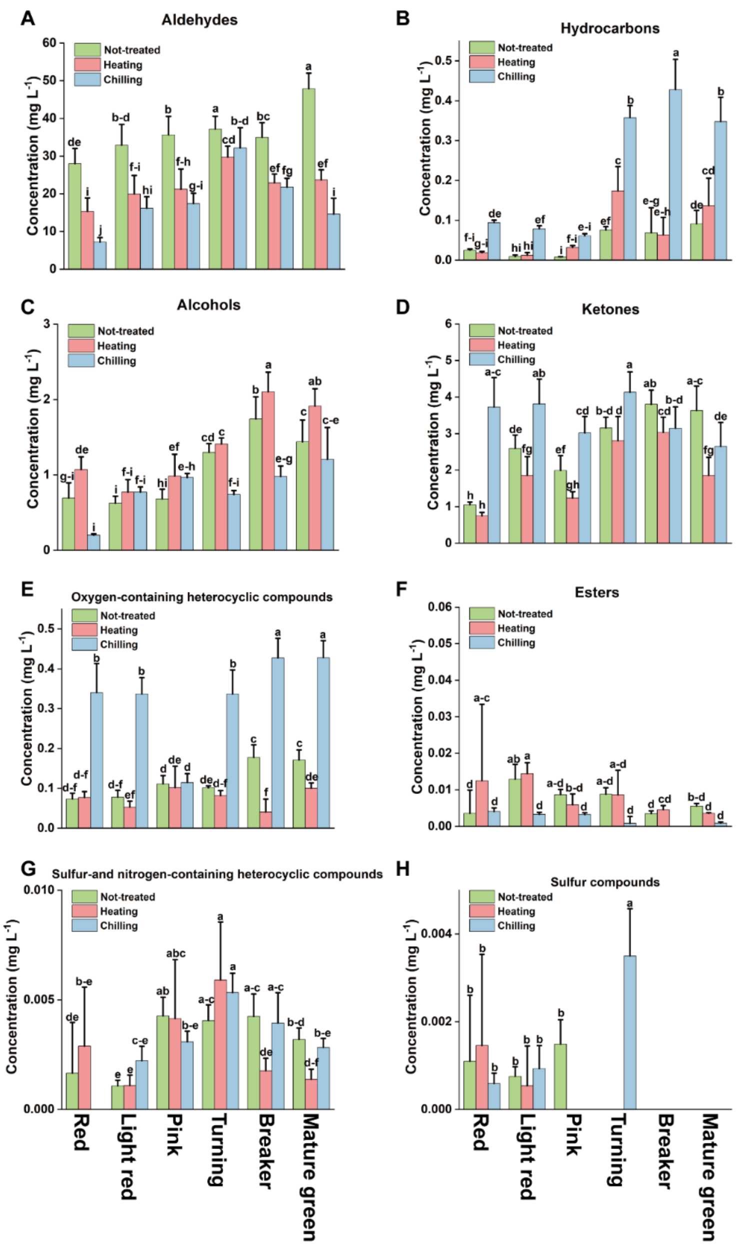

Figure S1. Effects of treatment combinations of harvest maturity and temperature on volatile concentration in tomatoes by chemical class: (A) aldehydes, (B) hydrocarbons, (C) alcohols, (D) ketones, (E) oxygen-containing heterocyclic compounds, (F) esters, (G) sulfur- and nitrogen-containing heterocyclic compounds, and (H) sulfur compounds. Each value is the mean of five replicates. Vertical bars represent standard deviation of the mean. Effect of “maturity × treatment” interaction was tested by two-way ANOVA ($p < 0.05$). Different letters on the top of columns represent significantly differences

between treatments using the Duncan Multiple Range test ($p < 0.05$).
