# Supplementary material for: Effects of Harvest Maturity, Refrigeration and Blanching Treatments on the Volatile Profiles of Ripe “Tasti-Lee” Tomatoes
Source: Foods. 2021 Jul 26;10(8):1727. doi: 10.3390/foods10081727 (PMC8391134; doi:10.3390/foods10081727)
Supplement: Supplementary file 1 [file foods-10-01727-s001.zip › Figure S2.pdf]

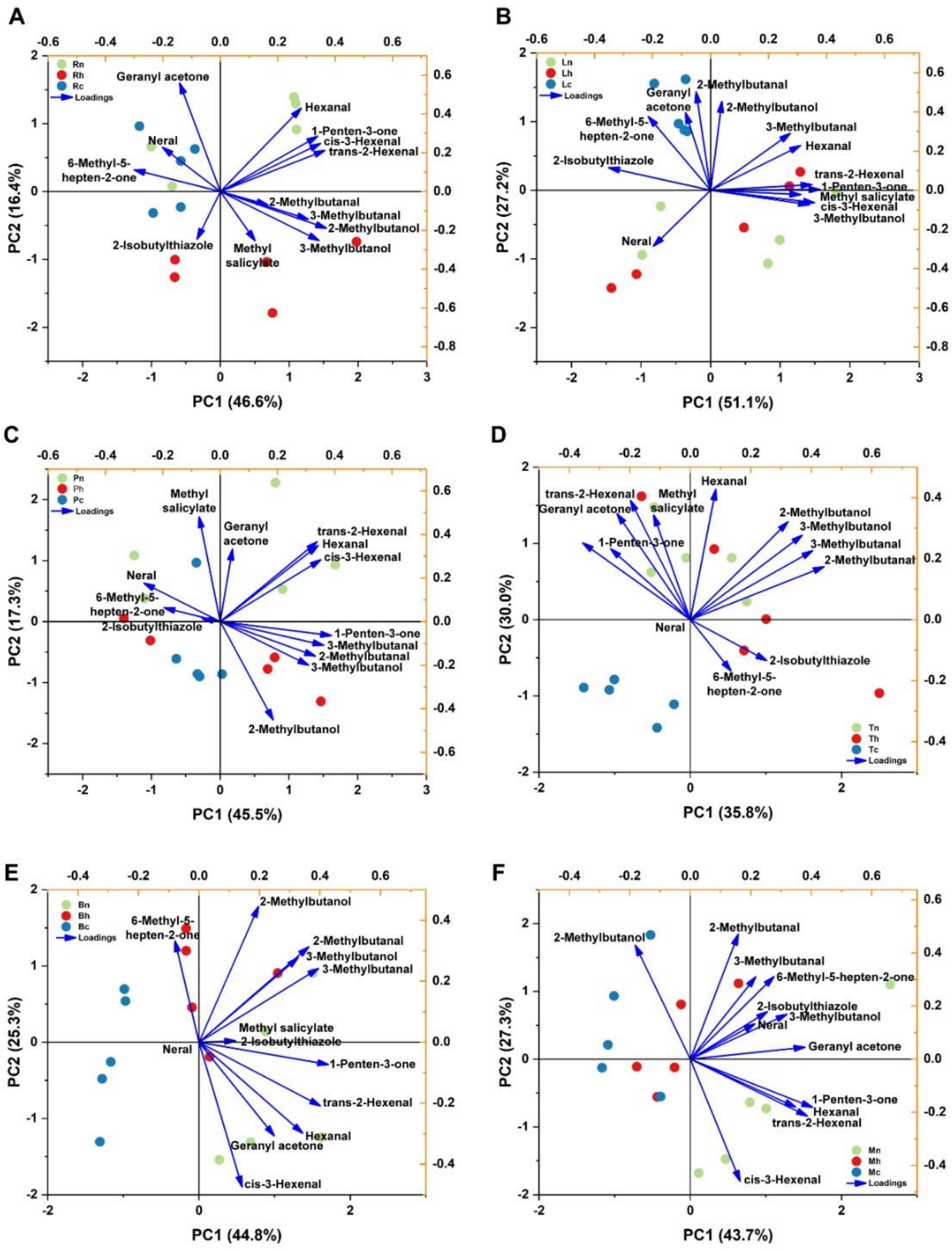

Figure S2. Principal component analysis (PCA) results based on the 13 key volatile substances in 'Tasti-Lee' tomatoes with different temperature treatments by six harvest maturities: (A) red, (B) light red, (C) pink, (D) turning, (E) breaker, and (F) mature green. Abbreviations represent combinations of harvest maturity (R – red; L – light red; P – pink; T – turning; B – breaker; and M – mature green) and temperature treatment (h – heating; c – chilling; n – non-treated control).
