# Supplementary material for: Effects of Harvest Maturity, Refrigeration and Blanching Treatments on the Volatile Profiles of Ripe “Tasti-Lee” Tomatoes
Source: Foods. 2021 Jul 26;10(8):1727. doi: 10.3390/foods10081727 (PMC8391134; doi:10.3390/foods10081727)
Supplement: Supplementary file 1 [file foods-10-01727-s001.zip › Table S1-revised.pdf]

Table S1. Relative concentration (%) of each chemical class determined in tomatoes harvested at six maturity stages and treated by refrigeration (chilling, c), blanching (heating, h) or non-treated (control, n)

Chemical class	Treatment (combination of harvest maturity and temperature treatment)																		Average
	Rn [z]	Rh	Rc	Ln	Lh	Lc	Pn	Ph	Pc	Tn	Th	Tc	Bn	Bh	Bc	Mn	Mh	Mc	
Aldehydes (%)	93.8	89.2	62.3	90.9	87.9	76.5	92.7	90.1	80.7	88.9	87.1	85.3	85.7	81.7	81.7	93.1	80.4	76.2	86.3
Hydrocarbons (%)	0.081	0.034	0.590	0.025	0.053	0.365	0.018	0.047	0.283	0.120	0.339	0.914	0.153	0.154	1.244	0.163	0.418	1.475	0.322
Alcohols (%)	2.341	6.059	1.736	1.704	3.400	3.665	1.770	4.154	4.475	3.109	4.122	1.961	4.298	7.361	3.654	2.788	6.497	6.284	3.677
Ketones (%)	3.530	4.211	32.416	7.091	8.291	17.876	5.177	5.250	14.001	7.585	8.171	10.946	9.378	10.628	11.781	3.602	12.276	13.809	9.053
Oxygen-containing heterocyclic compounds (%)	0.249	0.431	2.942	0.213	0.238	1.586	0.291	0.433	0.529	0.239	0.240	0.893	0.434	0.144	1.599	0.338	0.343	2.225	0.591
Esters (%)	0.013	0.067	0.035	0.035	0.066	0.014	0.023	0.025	0.014	0.019	0.026	0.003	0.007	0.014	0.000	0.008	0.017	0.005	0.020
Sulfur and nitrogen-containing heterocyclic compounds (%)	0.007	0.017	0.000	0.003	0.004	0.009	0.010	0.017	0.014	0.010	0.018	0.013	0.010	0.007	0.015	0.006	0.003	0.016	0.010
Sulfur compounds (%)	0.003	0.006	0.009	0.003	0.004	0.005	0.003	0.000	0.000	0.000	0.000	0.011	0.000	0.000	0.000	0.000	0.000	0.000	0.002

[z]: Abbreviations of combination of harvest maturity (R – red; L – light red; P – pink; T – turning; B – breaker; and M – mature green) and temperature treatment (h – heating; c – chilling; n – non-treated control).
